# Rheological properties and volumetric isothermal expansivity of bamboo kraft black liquor with high solids content and low lignin content

**DOI:** 10.1038/s41598-023-29350-0

**Published:** 2023-02-10

**Authors:** Shenglin Chen, Yongjian Xu, Kangkang Guo, Xiaopeng Yue

**Affiliations:** 1 College of Bioresources Chemical and Materials Engineering, Shaanxi University of Science and Technology, Xi’an, 710021 China; 2 National Demonstration Center for Experimental Light Chemistry Engineering Education, Shaanxi University of Science and Technology, Xi’an, 710021 China; 3 Key Laboratory of Paper Based Functional Materials, China National Light Industry, Shaanxi University of Science and Technology, Xi’an, 710021 China; 4 Shaanxi Key Laboratory On Paper Technology and Specialty Papers, Shaanxi University of Science and Technology, Xi’an, 710021 China

**Keywords:** Chemistry, Energy science and technology, Engineering, Fluid dynamics

## Abstract

In this study, a certain percentage of lignin in original bamboo kraft black liquor (BKBL) was separated, and the residual BKBL with low lignin content was expected to be fed into the alkali recovery boiler to reduce the heat transfer load of the alkali recovery boiler. With the decrease in lignin content, the rheological properties/volumetric isothermal expansivity (VIE) of BKBL change. When the lignin content was 70% remaining in the original BKBL, the viscosity of BKBL with low lignin content is close to that of the passivated BKBL at the same solids content, the dynamic viscoelasticity is superior, and the VIE decreases by 57.2%. When the amount of desilication agent is 1.5%, the viscosity of BKBL with low lignin content did not change much, and the VIE increased sharply and was 62.7% higher than that of the passivated BKBL. Therefore, the combination of partial lignin separation process and sodium aluminate desilication process can effectively improve the ability of alkali recovery boiler to deal with BKBL and reduce the influence of “silicon interference”.

## Introduction

The current situation in the Chinese pulp and paper industry is challenging as carbon emissions exceed the target, creating difficulty in realizing the goal of carbon peak and carbon net-zero^1–3^. Hence, it is imperative to realize the “integration of forest and pulp”. However, the “Silicon Interference” of non-wood pulping and the contradiction between the increase in pulp demand and the insufficient capacity of the existing alkali recovery system restrict the development of pulp paper integration. Improving pulp production capacity is the key to expand the treatment capacity of alkali recovery system and reduce the impact of “Silicon Interference”^4–8^. Building a new alkali recovery system is the most direct and effective method, but is bound to increase the production cost of the pulp mill. If the black liquor from a new pulp production line shares the original alkali recovery system with the black liquor from the existing production lines, the expansion of pulp production capacity may become a reality. There is about 25% of the lignin in bamboo material, which enters the bamboo kraft black liquor (BKBL) during cooking. Especially, lignin (0.5 tons) will enter the BKBL with the production of 1 ton of bamboo pulp. Lignin is a high-quality renewable raw material that can be used as a substitute for many petroleum-based products. Therefore, it is useful to separate a certain percentage of lignin from BKBL as a byproduct^9–14^. The revenue from the sales of lignin brings additional economic benefits to the pulp mill, which can be used to cover the upfront investment in building a lignin separation unit. At the meantime, the residual black liquor could be still pumped to the alkali recovery system. This can reduce the heat exchange load of alkali recovery boiler and improve the treatment capacity of black liquor^15–18^. In addition, it has a certain desilication effect when separating lignin due to the adsorption of silicon by lignin, which is helpful for overcoming the “Silicon Interference”^19,20^.

The problem of “Silicon Interference” in non-wood pulping is very serious, and the extraction of some lignin cannot completely solve it. In view of this situation, our research group proposed the process of “desilication from green liquor by BKBL combustion”. Desilication agent is added before the BKBL enters the recovery boiler, and the green-liquor-silicon-insoluble (GLSI) substance is formed in the combustion stage. The desilication purpose from green liquor can be achieved by filtering the GLSI substance, and the white mud with low silicon content can be recycled. The alkali recovery circulation system can form a closed loop. Therefore, the influence of desilication agent on the rheological and isothermal expansion properties of BKBL also needs to be considered. The extracted lignin is beneficial to the flow and may hinder the isothermal expansion characteristics (VIE) of black liquor^21–24^. Therefore, the proportion of extracted lignin is very important and needs to achieve a balance between the rheological and isothermal expansion properties. Specific experimental data must be provided to support whether it can replace the high-temperature passivation section or reduce the passivation treatment time based on the declining degree of viscosity, and whether the adverse impact on the expansion performance can be tolerated in actual production process.

The rheology and VIE of BKBL with low lignin content and high solids content were systematically studied. We analyzed the rheological parameters, the changes in apparent viscosity η_a_, storage modulus G′, dissipation modulus G″, and the dynamic viscosity η′ of high solids content BKBL. The VIE value of BKBL was measured and its variation pattern was analyzed. The promoting or inhibiting effect of extracted lignin on rheological parameters was investigated, and the improving or hindering effect of adding desilication agent on isothermal expansion properties was also discussed.

## Experimental

### Materials

The BKBL from Kraft pulping in G2 tower continuous digester was provided by Guizhou Chitianhua Co., Ltd, China. The relative density was 1.21 g/cm^3^, the total solid content was 475.00 g/L, the organic content was 270.88 g/L, and the inorganic content was 204.12 g/L. The organic components of BKBL mainly include lignin and hemicellulose, while the inorganic components include Na_2_SO_4_, Na_2_SiO_3_, SiO_2_, and Si (OH)_4_. Sulfuric acid (H_2_SO_4_, AR, 98%) was purchased from Sinopharm Chemical Reagent Co., Ltd. Aluminum sulfate (Al_2_(SO_4_)_3_, AR, 99.8%) provided by Damao Chemical Reagent Factory Co., Ltd. Partial sodium aluminate (NaAlO_2_, AR, 99.8%) was obtained from Sinopharm Chemical Reagent Co., Ltd.

### Preparation of BKBL with high solid content and low lignin content

**Figure 1:**
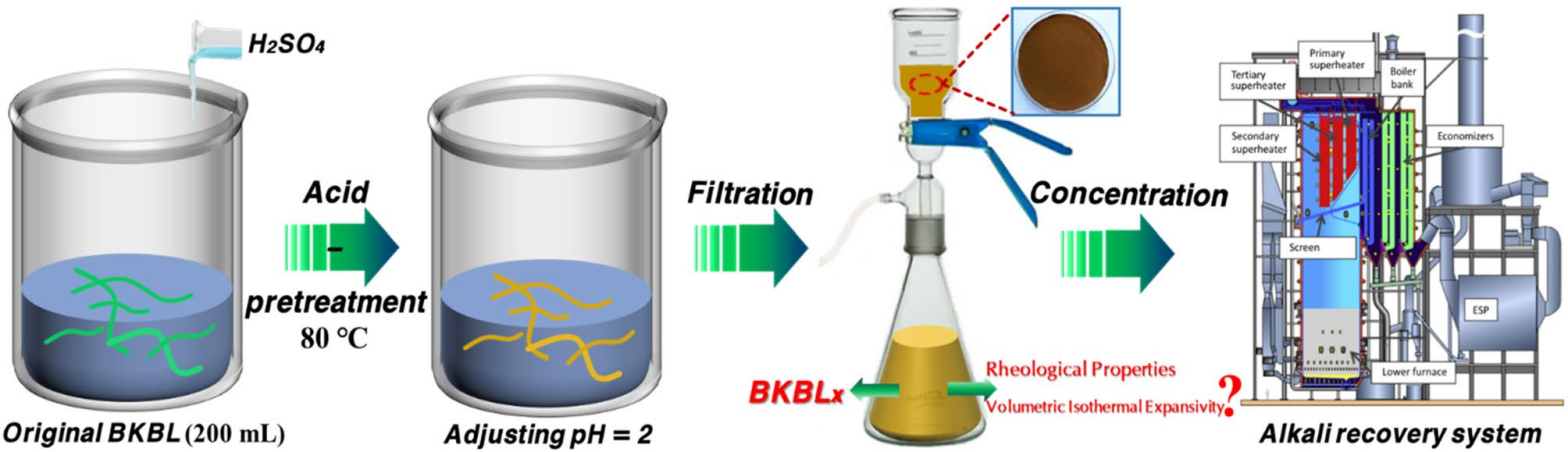
Schematic diagram of preparation process of BKBL_X_.

The preparation process of BKBL with low lignin and high solids content was shown in Fig. 1. The original BKBL (200 mL) was placed in a beaker, and the H_2_SO_4_ was added into the beaker, adjusting continuously until the pH reaches to 2. The acidified BKBL was left and precipitated for 1 h at 80 °C, and all the precipitated lignin was filtrated out. The filtered lignin was washed with distilled water and dried to a constant weight in an oven at 105 °C, and the lignin content of original BKBL was calculated. According to the above method, the pH of original BKBL was adjusted slightly with sulfuric acid in order to separate 10%, 20% and 30% lignin (present the percentage of total lignin) from the BKBL, respectively. Therefore, the residual BKBL has low lignin content with 70%, 80% and 90% lignin compared to the original BKBL, respectively. Then all the BKBL samples were concentrated to 80 wt% at 80 °C in a vacuum drying oven, which named BKBL_x_ (e.g., BKBL_70_ express that the lignin content is 70% based on total lignin in the original BKBL). In addition, the effect of adding desilication agent on the rheological properties was studied. BKBL_70_ was divided into three sections, with 0%, 0.5% and 1.5% of the desilication agent NaAlO_2_ added into the three sections, respectively.

### Instrument and characterization

#### Rheological properties

The rheological parameters of BKBL were tested by using a dynamic rheometer (AR2000ex, TA, American). The test was carried out using a 25 mm diameter copper parallel plate fixture with a spacing of 700 µm between the upper and lower parts of the fixture. The range of shear rate was varied from 0 to 100 s^−1^ in the apparent viscosity test, and the vibration frequency was set at 0–105 rad/s in the dynamic viscoelasticity test.

#### Volumetric isothermal expansivity

The VIE of BKBL was tested in a muffle furnace (KSY15-16, Yifeng Electric Furnace Co., Shanghai, China). 2.5 g of BKBL were weighted with corundum crucible and put into muffle furnace, and the muffle furnace was then heated from room temperature to 300 °C and kept for 1 h in order that the black liquor is carbonized and expands fully. The volume of carbonized black liquor in corundum crucible with dry particles was measured. The ratio of volume to mass was obtained as the VIE of BKBL.

## Results and discussion

### Effect on the apparent viscosity of BKBL_X_

Zamanet al. found black liquor with high solids content is non-newtonian fluid. Its viscosity and viscoelasticity increase with the increase of solid content. In addition, he used “Power-law” to fit the viscosity changes within the test range, estimated the viscosity value and viscoelastic modulus value of black liquor at higher solid content and temperature through these models^25–27^. Sinquefield found that the viscosity of wood pulp black liquor with solid content of 10–30% increased with the increase of solid content^28^. Jawaid et al. found that the viscosity of medium-concentration wood pulp black liquor (note: solids content 37–47%) increased with the increase of solid content^29^. Wallmo found that when the extraction amount of lignin was 60%, the viscosity of black liquor decreased by 75% (note: solids content 50%)^30^. Moosavifar et al. found that after the removal of lignin, the viscosity can be reduced by one order of magnitude at most, and the boiling point of black liquor will also decrease correspondingly^31^. In the above studies, the object base is wood pulp black liquor. Therefore, it is necessary to study the black liquor of non-wood pulp. Zhang and Sun studied the dynamic viscoelastic characteristics of BKBL with medium concentration and the effect of desilication agent^32,33^. In order to improve the treatment capacity of black liquor, they concluded that future trend is to separate some lignin from black liquor before being sent it to alkali recovery boiler for combustion. However, attention should be paid to the dynamic viscoelasticity of BKBL_X_ with low lignin content and high solid content.

#### Effect of lignin content on the apparent viscosity of BKBL_X_

**Figure 2:**
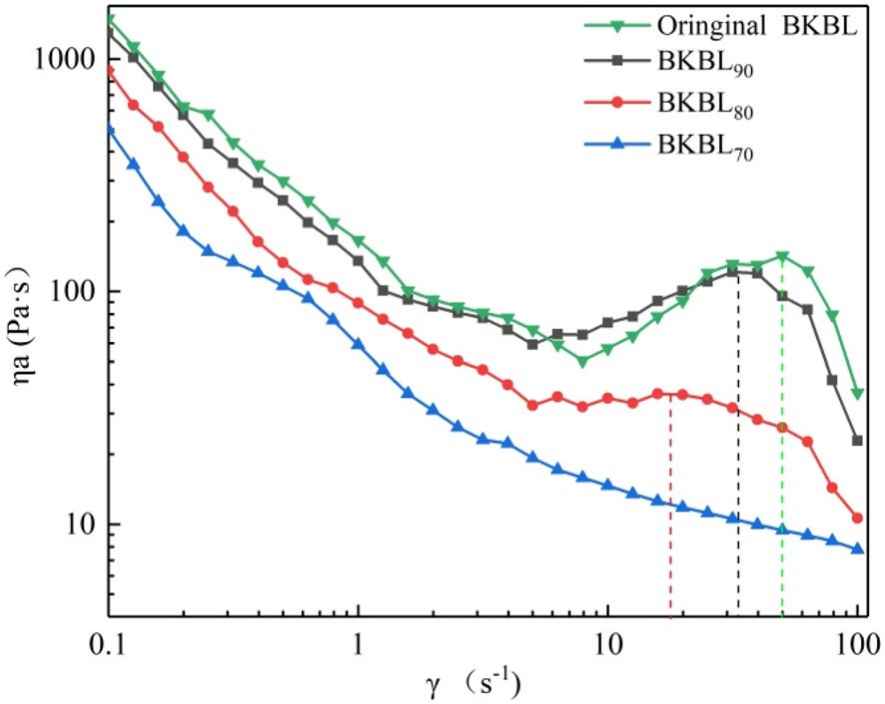
η_a_ versus γ for BKBL_X_ (Note: 80 wt%, 98 °C).

The viscosity of BKBL affects the pump transportation and evaporation efficiency. The results of the apparent viscosity of BKBL_x_ were shown in Fig. 2. The apparent viscosity of original BKBL and BKBL_x_ decreases significantly with the increase in shear rate when 98 °C. The curve of BKBL_90_ and BKBL_80_ are similar to that of original BKBL, but the viscosity and thickening rate decreased with the decrease in lignin content. The apparent viscosity of BKBL_70_ maintains a downward trend within the testing range, and there is no shear thickening observed. The main reason is the decrease in the strength of network structure formed in BKBL_70_ because of the significant reduction of lignin content in BKBL_70_, which could be destroyed without high shear. Compared with the previous work in our group, it is found that the apparent viscosity of BKBL_70_ is approximately the same as that of the passivated BKBL at the shear rate of 100 s^−1^ when they have the same solid content. Therefore, the purpose of viscosity reduction may be achieved by extracting some lignin in commercial production process, and the passivation section may be eliminated in the alkali recovery system or the passivation time may be shortened, so as to effectively reduce the energy consumption in alkali recovery system.

The reason why the reduction of lignin content helps to reduce the viscosity of black liquor is that in the process of acid precipitation, lignin is separated step by step. The lignin with the largest molecular weight was first separated. The strength of the network structure formed by lignin decreases, when the content of lignin decreases, which is manifested by the decrease of the viscosity of black liquor.

**Figure 3:**
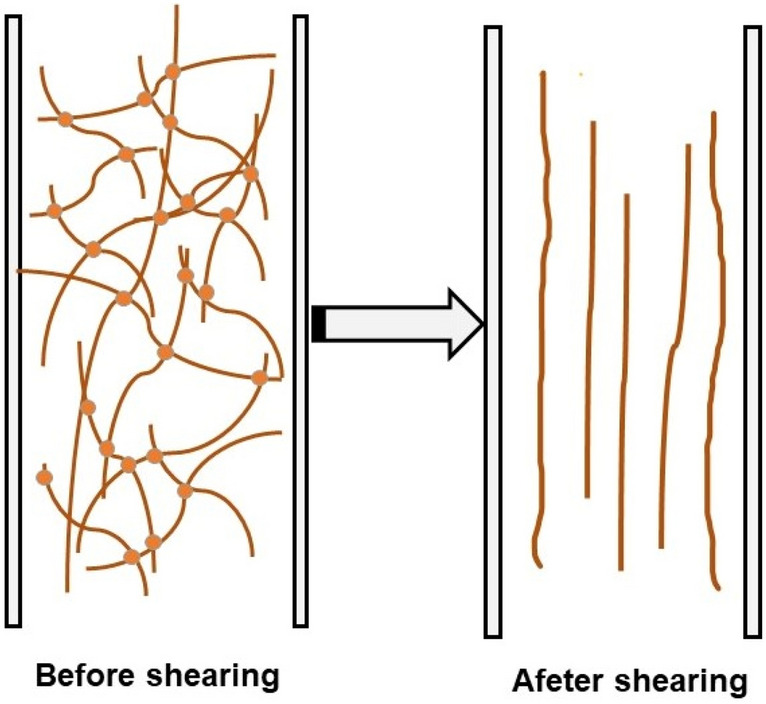
Orientation change of lignin in BKBLx under the shear flow field.

The shear thinning of BKBL apparent viscosity can be explained by “polymer conformational change theory”. BKBL with high solids content only contains a small amount water, and there exist organic macromolecules including lignin in the form of long linear chains. As long as the length of some molecular chains is greater than a certain value, the molecular chains will entangle with each other and form entangled floccules somewhere in the black liquor. The number of entangled floccules is in a dynamic equilibrium, and a network structure may be formed with the polymers in the black liquor when the equilibrium is broken. Some energy is stored in the internal network during the BKBL flow, and the external performance is that BKBL has high viscosity and is difficult to flow. As BKBL is subjected to continuous shearing, the direction of its internal disordered molecular chains is gradually consistent with the shear force (as shown in Fig. 3). At this time, the shear effect is directly used for the flow of BKBL, and the external performance is a sharp decline in the apparent viscosity of BKBL.

#### Effect of desilication agent and temperature on the apparent viscosity of BKBL_70_

**Figure 4:**
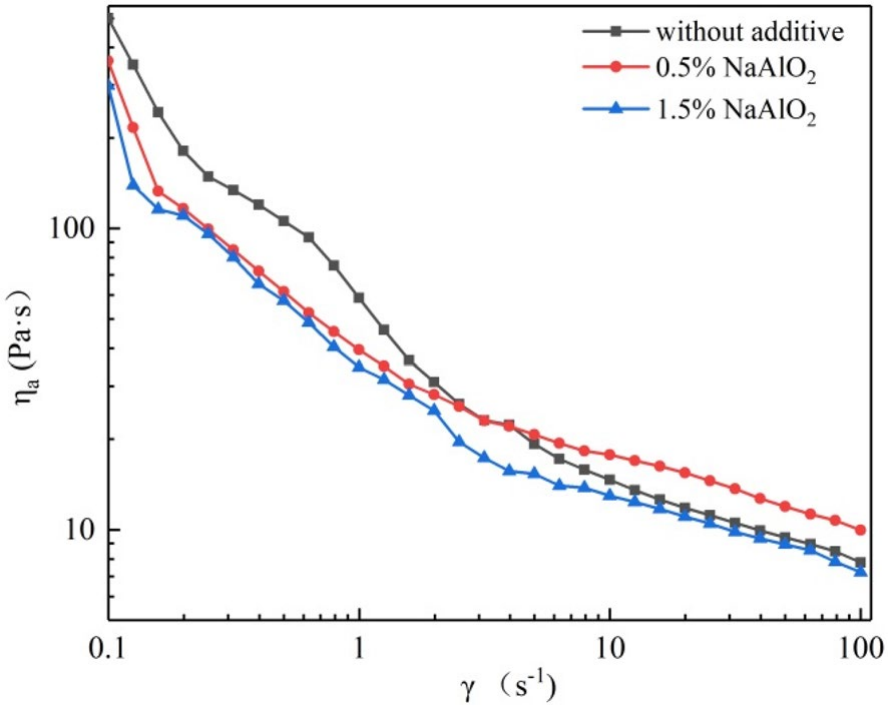
Relationship between η_a_ versus γ for BKBL_70_ with the loading of sodium aluminate (Note: 80 wt%, 98 °C).

Previously, some researchers studied the desilication effect of aluminum desilication agent, magnesium desilication agent, boron desilication agent and composite desilication agent on wheat straw pulp BKBL. The research mainly focused on the desilication efficiency of the desilication agent, and the rheological properties of the original BKBL and the desilicated BKBL have not been studied. Our research team studied the effect of desilication agent on the apparent viscosity of BKBL with high solid content, and the BKBL_70_ is chosen as the object to study the influence of desilication agent, the results were shown in Fig. 4.

It could be seen in Fig. 4 that the desilication agent had a certain positive effect on the reduction in viscosity. The desilication agent NaAlO_2_ contains Na^+^, and Na^+^ can combine with the lignin macromolecules in the black liquor, which increases the electrostatic repulsion force between the lignin macromolecules. What’s more, NaAlO_2_ is alkaline. Long-chain lignin breaks into short-chain lignin under the action of OH^−^. The strength of molecular structure formed by the long chain of lignin decreases, and the viscosity of black liquor decreases.

**Figure 5:**
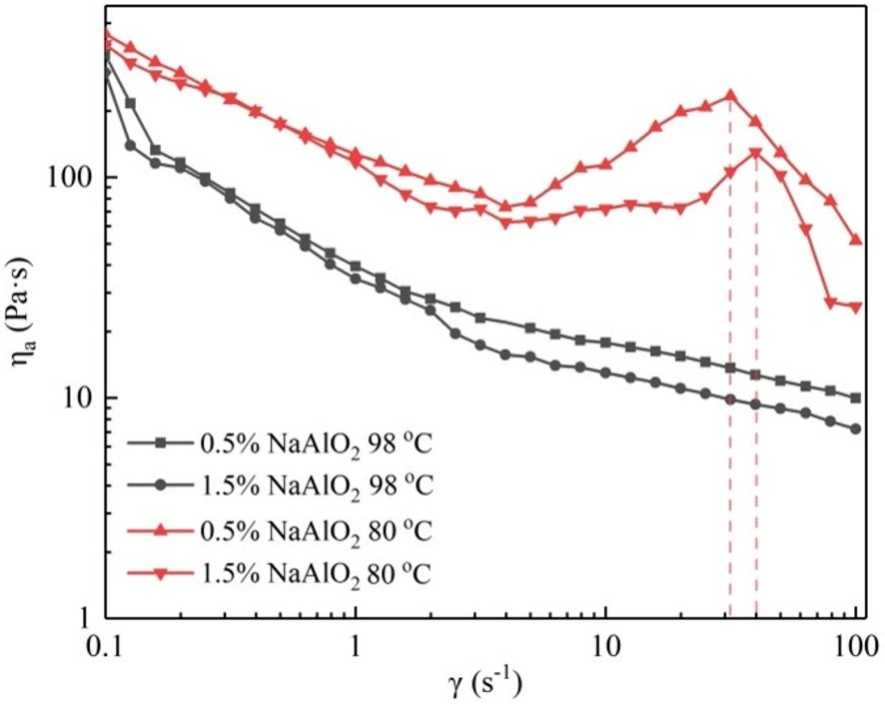
η_a_ versus γ for BKBL_70_ at different temperatures (Note: 80 wt%, BKBL_70_).

The relationship between η_a_ and. γ was revealed in Fig. 5. It can be seen from Fig. 5 that BKBL_70_ samples with desilication agent show shear thickening at 80 °C, while not at 98 °C. Combined with Figs. 2 and 3, it infers that not only lignin content but also temperature affect shear thickening, and the shear thickening is caused by the long lignin molecular chain entanglement. In our opinion, the uniform orientation of lignin molecular chains under shear force is not the only explanation for shear thinning of BKBL, the rubber-like liquid theory is also applicable to explain the shear thinning phenomenon of BKBL. Under the theory, the molecular chains in black liquor could produce the entanglement behavior which is mainly controlled by molecular thermal motion. The rate at which entanglement floccules are destroyed by shearing or heating is greater than the rate at which they are generated.

In fact, it is impossible to increase the temperature of black liquor indefinitely. When the solid content reaches a certain limit, the effect of temperature on viscosity is not the main factor. Therefore, viscosity can only be further reduced by separating part of lignin. The most important thing is that separating part of lignin can improve the ability of treating black liquor by reducing the heat load of alkali recovery boiler.

### Effect on the dynamic viscoelasticity of BKBL_x_

#### Effect of lignin content on the dynamic viscoelasticity of BKBL_x_

Storage modulus G′, also known as elastic modulus, refers to the energy stored due to elastic (reversible) deformation of the materials during deformation, reflecting the elastic-solid property of the materials. Loss modulus, G″, i.e., viscosity modulus, refers to the energy lost due to viscous deformation (irreversible) of materials during deformation, reflecting the viscous flow of the materials.

**Figure 6:**
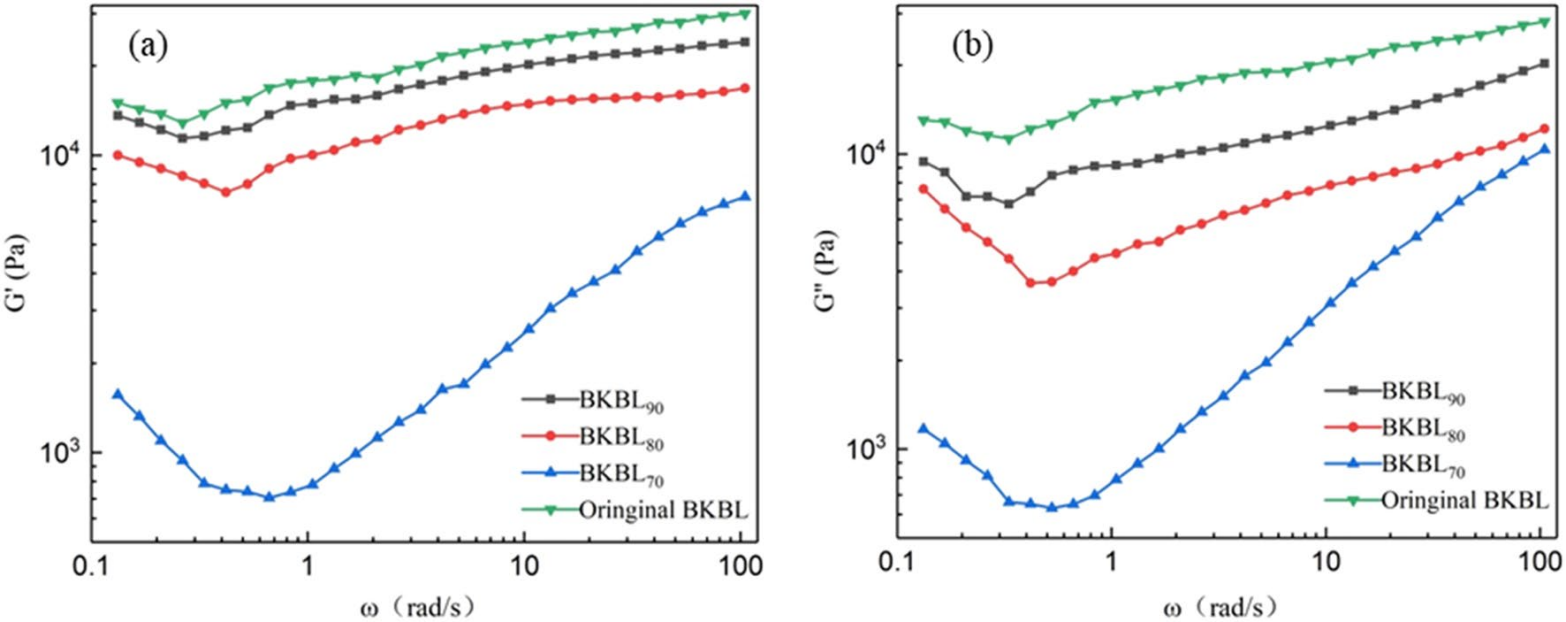
G′ and G″ versus ω for BKBL_X_ (Note: 80 wt%, 98 °C).

As can be seen from Fig. 6, the G″ and G″ of BKBL_x_ first decrease slightly and then increase with the increase in oscillation frequency. Moreover, the G″ is always higher than the G″, indicating that the reversible deformation is greater than the irreversible deformation for BKBLx. The decrease in lignin content is responsible for the decrease in the G′ and G″, indicating that the viscoelasticity decreases. Even when BKBLx encounters elbows, valves, nozzles and other structures in the pumping process, the movement obstacles encountered by BKBLx are far less than those encountered by the original BKBL in the normal straight pipe transportation.

#### Effect of lignin content on the dynamic viscosity η′ of BKBL_X_

**Figure 7:**
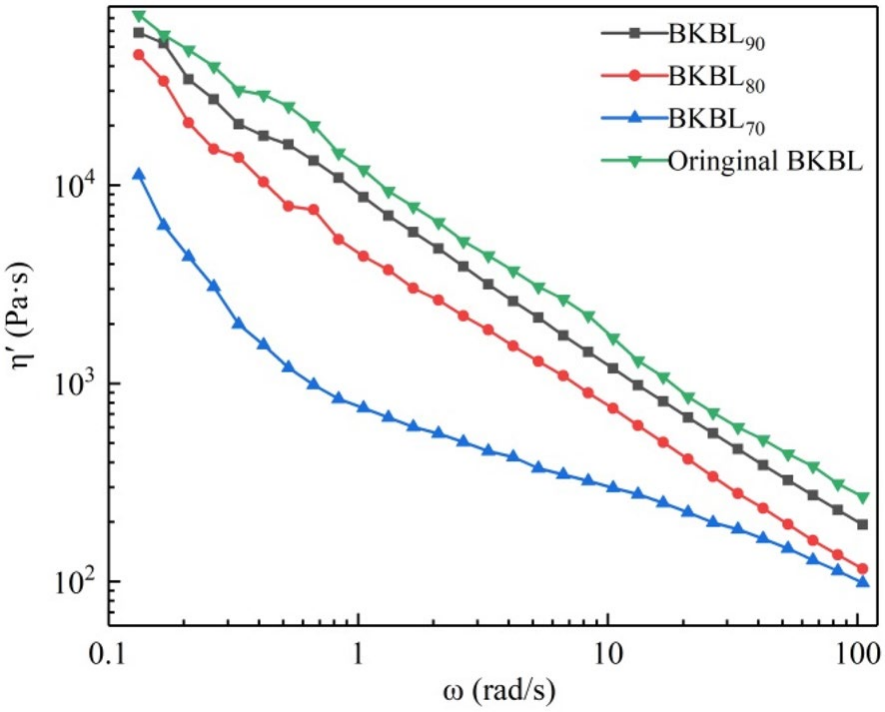
Relationship between η′ versus ω for BKBL_X_ (Note: 80 wt%, 98 °C).

Dynamic viscosity η′ refers to the change rate of loss modulus G″ with angular frequency ω. The dynamic viscosity can eliminate the measurement error of apparent viscosity caused by elastic deformation during the flow of some BKBL samples, so we measured the dynamic viscosity to evaluate the fluidity of the black liquor. Figure 7 shows the effect of lignin content on dynamic viscosity η′ of BKBL. It can be seen from Fig. 7 that the η′ curves of BKBL_x_ and original BKBL show the trend of shear thinning, which is similar to the trend of apparent viscosity η_a_. This further proved that the separation of some lignin is benificial to the pumping and transportation of BKBL_x_.

#### Effect of desilication agent on the G′ and G″ of BKBL_70_

**Figure 8:**
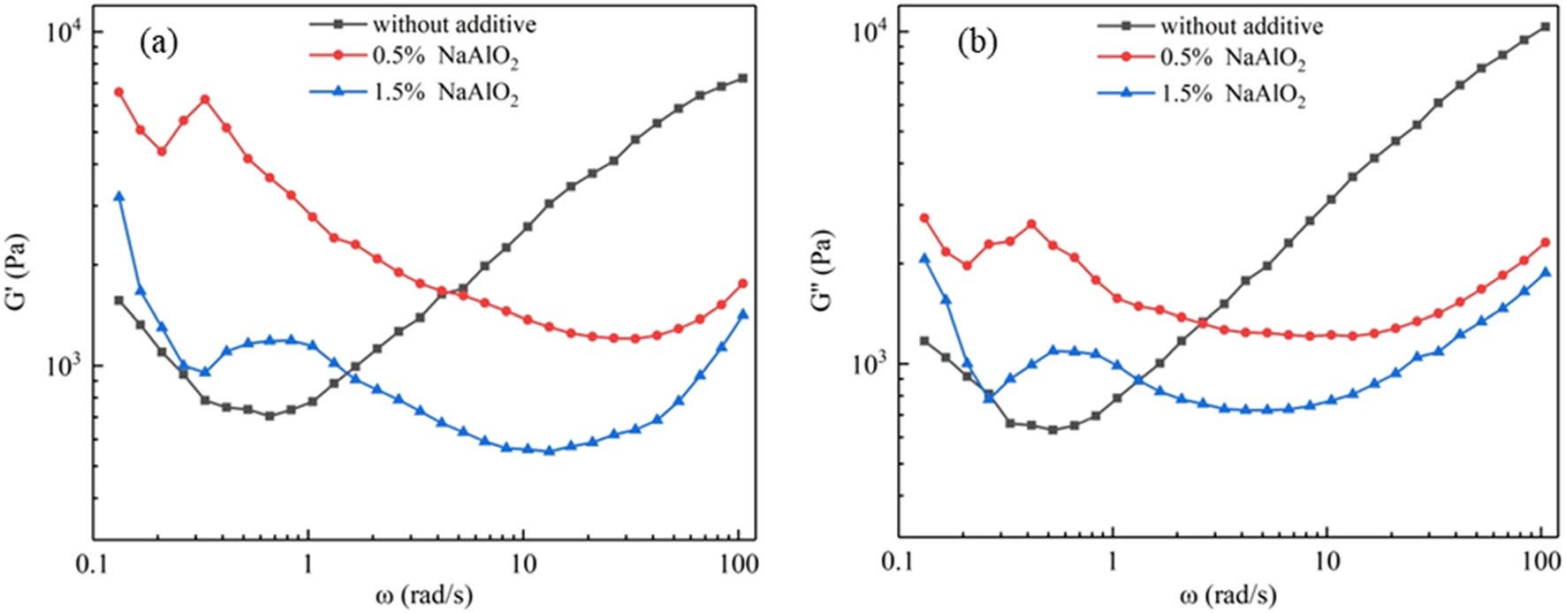
G′ and G″ versus ω for BKBL_70_ with the loading of sodium aluminate (Note: 80 wt%, 98 °C).

It can be seen from Fig. 8 that the G′ and G″ of BKBL_70_ increased when NaAlO_2_ was added at zero oscillation frequency. With the increase in oscillation frequency, G′ and G″ decreased rapidly at first, and then started to increase when the oscillation frequency was greater than 10 rad/s. The viscoelastic modulus of BKBL_70_ with adding desilication agent was less than that of the original BKBL_70_. That is to say desilication agent has little adverse effect on viscoelasticity of black liquor at high oscillation frequency.

#### Effect of desilication agent and temperature on the dynamic viscosity η′ of BKBL_70_

**Figure 9:**
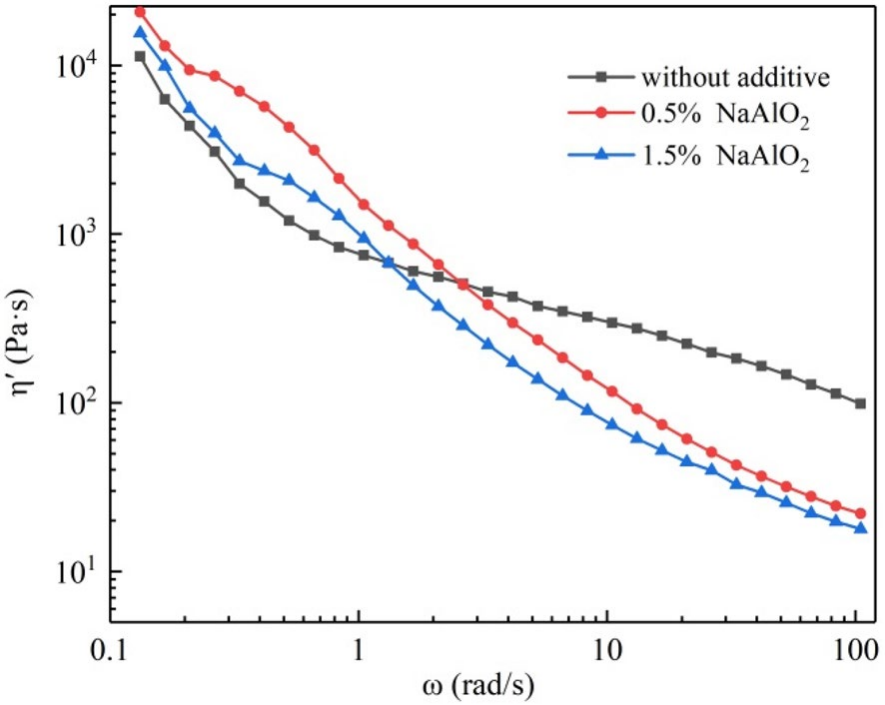
η′ versus ω for BKBL_70_ with the loading of sodium aluminate (Note: 80 wt%, 98 °C).

It can be seen from Fig. 9 that the variation pattern of BKBL_70_ dynamic viscosity η′ with angular frequency is similar to that of the apparent viscosity η_a_, and they all show a trend of shear thinning. At low oscillation frequency, desilication agent leads η_a_ to increase, indicating that the viscous kinetic energy loss of BKBL increases at this time. The apparent viscosity of BKBL samples decreases when adding NaAlO_2_ desilication agent, which is consistent with to the previous results.

As for BKBL_70_, the reason why the dynamic viscosity with 0.5% NaAlO_2_ is greater than that with 1.5% NaAlO_2_ is that the viscosity of the former is greater than that of the latter, and the kinetic energy loss caused by high viscosity is also greater. When the angular frequency is close to 100 rad/s, the dynamic viscosity of BKBL_70_ with 1.5% desilication agent is lower, because the dynamic viscosity is closer to the actual viscosity than the apparent viscosity at high oscillation frequency. This shows that the viscosity of BKBL_70_ decreases with the increase in the amount of desilication agent.

**Figure 10:**
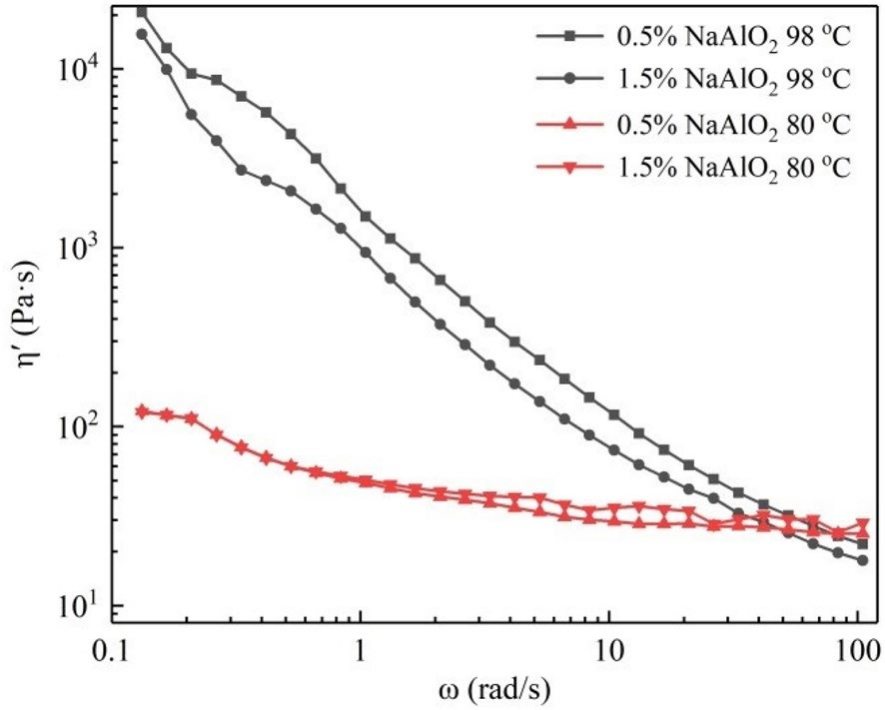
η′ versus ω for BKBL_70_ at different temperatures (Note: 80 wt%, BKBL_70_).

Figure 10 shows the effect of temperature on the dynamic viscosity. At low oscillation frequency, the dynamic viscosity of BKBL_70_ at 80 °C is much lower than that at 98 °C, indicating that BKBL_70_ is mainly elastic-solid at 80 °C. Dynamic viscosity tends to be the real viscosity with the increase in angular frequency, and the reason is that the main role of stress is to make BKBL_70_ undergo elastic deformation. The dynamic viscosity of BKBL_70_ decreases rapidly at 98 °C and is slightly lower than that at 80 °C when the oscillation frequency is close to 100 rad/s. This further shows the importance of high temperature for BKBL transport.

The shearing effect and stress on BKBL in pipeline transportation and nozzle atomization are much higher than those in the lab testing process. Therefore, it is reasonable to speculate that adding NaAlO_2_ desilication agent will cause little adverse effect on viscosity but a certain viscosity reduction effect when BKBL_70_ are pumped into the alkali recovery boiler.

### Effect of lignin content and desilication agent on VIE of all BKBL samples

Papermakers can judge the difficulty of concentration and dryness of black liquor in the boiler and predict the swollen degree of the bottom cushion layer according to the VIE of black liquor. The larger the VIE value, the more swollen the bottom bed, and the easier the air goes through the bed, the better the actual combustion effect. The VIE of straw pulp black liquor is the smallest among the various raw materials black liquor because of its high silicon content and high viscosity. Therefore, it is essential to ensure that the VIE of BKBLx is greater than that of straw pulp black liquor, so that a good bed can be formed at the bottom of alkali recovery boiler.

**Figure 11:**
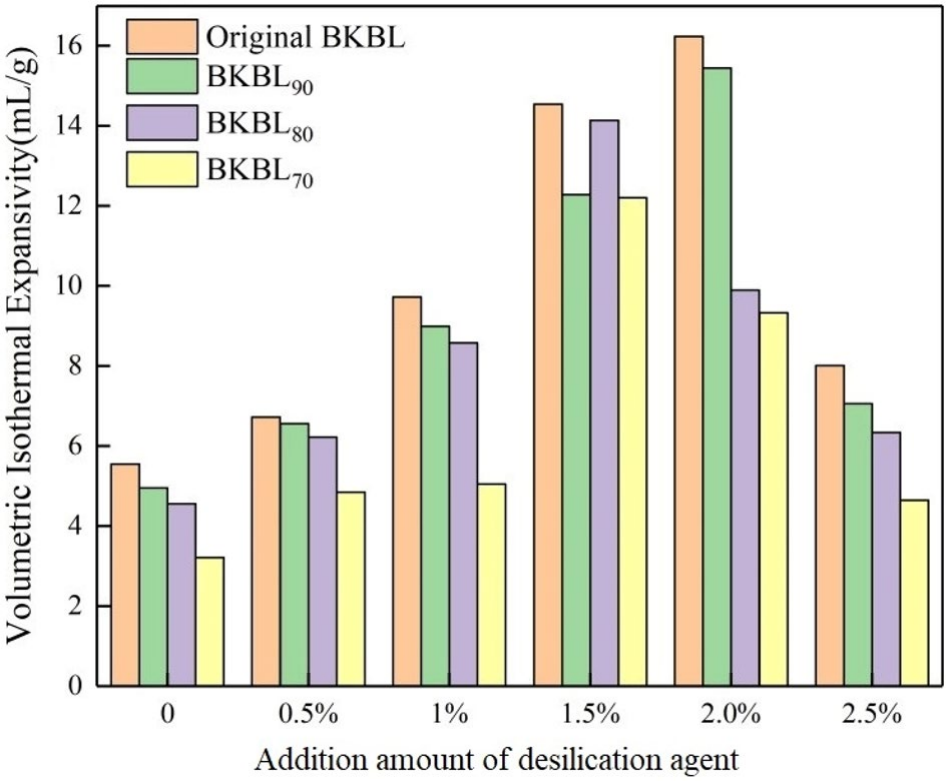
VIE of BKBL_x_ with the loading of desilication agent.

It was indicated that the effect of lignin content and NaAlO_2_ VIE of BKBL were measured by VIE of BKBLx in Fig. 11. Without adding desilication agent, the VIE of BKBL_x_ decreases with the decrease in lignin content, and the VIE of original BKBL is almost twice that of BKBL_70_, indicating that the lignin content has a great influence on the VIE of BKBL_x_, which is due to the release of gas during the thermal decomposition of lignin. It is concluded that the reason for the decrease of VIE is the decrease in gas emissions due to the decrease in lignin content in BKBL_x_^34^. Although the decrease in lignin content directly leads to the decrease in the VIE of BKBL_x_, the increase in VIE will be more obvious in the presence of promoting factors. Impact on NaAlO_2_ desilication agent on VIE may be caused by the viscosity reduction effect of NaAlO_2_. It can be seen from the previous research that the addition of NaAlO_2_ can effectively reduce the apparent viscosity of BKBL at zero shear rate and improve the elastic modulus of BKBL at zero oscillation frequency. Therefore, adding a small amount of desilication agent can significantly promote the VIE of BKBL.

**Figure 12:**
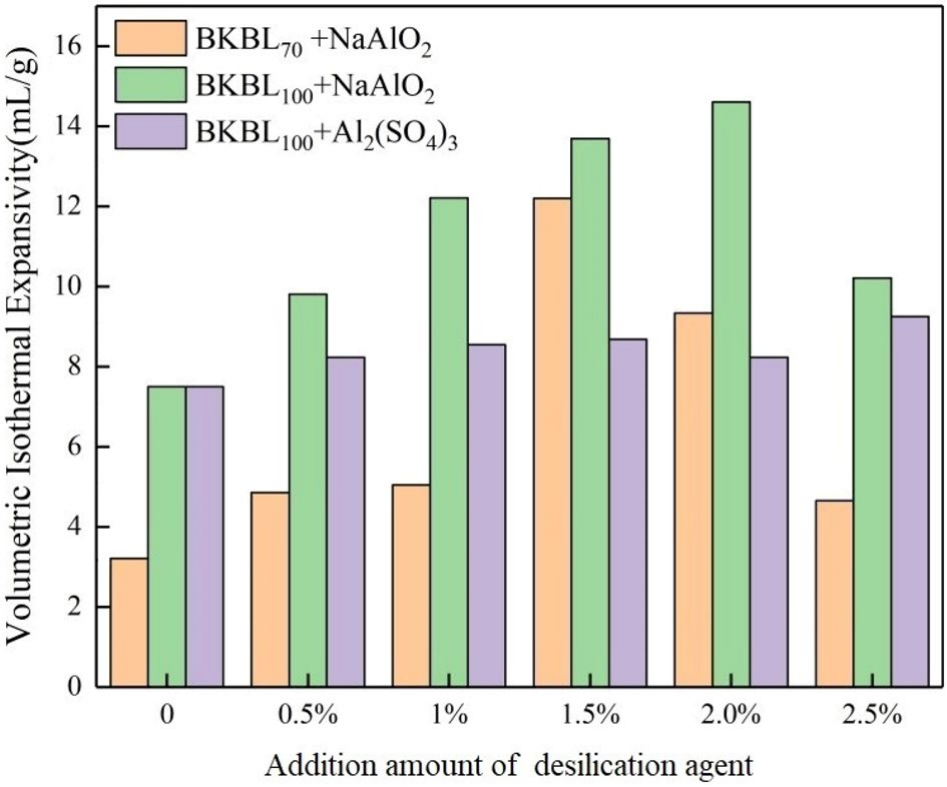
Promoting effect of desilication agent on VIE of BKBL_70_. The amount of desilication agent (based on total lignin content in BKBL).

The changes of the VIE of passivated BKBL and BKBL_70_ with different desilication agent are shown in Fig. 12. NaAlO_2_ has clear improving influence on VIE due to its viscosity reduction, and acidic desilication agent Al_2_(SO_4_)_3_ has no significant effect. The reason is that Al_2_(SO_4_)_3_ leads to the increase in the viscosity and elastic solid properties of BKBL, and the improvement for elastic solid properties and hindering effect for the increase in the viscosity can offset each other. Therefore, the VIE of BKBL will not change significantly. The VIE of BKBL_70_ with alkaline NaAlO_2_ desilication agent added was 62.72% higher than that of passivated BKBL. Furthermore, a small amount of desilication agent can reduce the adverse effect of extracted lignin on the expansion performance of BKBL. In this work, the desilication rate is the highest and meanwhile the VIE value reaches the maximum, when the amount of desilication agent is 1.5%.

In brief, the separation of some lignin can not only greatly decrease the viscosity of BKBL, but also reduce its adverse effect on the expansion performance by adding desilication agent. What’s more, the combined use of “Partial Lignin Separation Process” and “Desilication from Green Liquor through Black Liquor Combustion Process” can improve the processing capacity of BKBL and ultimately helping to improve the production capacity of pulp.

## Conclusions

In order to improve the ability of alkali recovery boiler to treat BKBL and reduce the impact of “Silicon Interference”, a certain percentage of lignin was separated from BKBL, and we intend to send the remaining BKBL with low lignin content to the existing alkali recovery boiler. Therefore, we studied the rheological and VIE of BKBL with high solid and low lignin content, and the effect of desilication agent on them. The viscosity and VIE of BKBL are closely related to lignin content. With the decrease in lignin content, the apparent viscosity and VIE of BKBL_X_ decrease. When 30% lignin was separated from the original BKBL, the apparent viscosity of the remaining black liquor-BKBL_70_ was close to that of the passivated BKBL with the same solids content, and the storage modulus and dissipation modulus were lower than those of the original BKBL. The VIE of BKBL_70_ is smaller than that of the passivated BKBL with the same solids content by 57.2% BKBL_70_ has a trend of transition from non-Newtonian fluid to Newtonian fluid, but the VIE declines too much, which induce that BKBL_70_ cannot form a cushion with a good intumescent performance at the bottom of the alkali recovery boiler. The adverse influence on decreasing lignin content of VIE can be compensated by adding NaAlO_2_ desilication agent, and the VIE of BKBL_70_ is significantly increased with adding 1.5% NaAlO_2_ desilication agent by 62.7% higher than that of passivation black liquor. In addition, the apparent viscosity of BKBL decreases with adding a small amount of NaAlO_2_ desilication agent, and the rheological properties of BKBL_70_ are not be clearly affected, which is beneficial to the pumping and transportation of BKBL.

## Data Availability

The data used and/or analyzed during the current study are available from the corresponding author on reasonable request.
